# Differential Impacts of Perfluorooctanoic Acid (PFOA) on Soil Microbial Communities in Aerobic and Anaerobic Agricultural Soils

**DOI:** 10.1021/acsomega.5c06624

**Published:** 2025-09-19

**Authors:** Nusrat Easmin, Parikrama Sapkota, Kelly S. Ramirez, Yasaman Mohammadi, Mahesh Narayan, Hamidreza Sharifan

**Affiliations:** † Department of Chemistry and Biochemistry, 12337 University of Texas at El Paso, El Paso, Texas 79968, United States; ‡ Environmental Science and Engineering Program, University of Texas at El Paso, El Paso, Texas 79968, United States; § Department of Biological Sciences, University of Texas at El Paso, El Paso, Texas 79968, United States; ∥ Sharifarm LLC, Research & Development, El Paso, Texas 79911, United States

## Abstract

Perfluorooctanoic acid (PFOA) is a persistent environmental contaminant with the potential to disrupt soil-plant systems. This study investigated the impacts of PFOA on soil microbial viability and community composition under aerobic and anaerobic conditions, to understand microbial resilience and adaptability to pollutant stress. Soil samples from an uncontaminated agricultural field (2.26 ng/g) were amended with varying PFOA concentrations (0, 40, 60, 80, and 100 mg/kg) and incubated under controlled conditions for 7 days. Microbial viability was assessed through colony-forming unit (cfu) counts, while community composition was characterized via 16S rRNA gene sequencing. Results showed a significant decline (8–79%) in microbial viability at higher PFOA concentrations across both environments, with anaerobic soils exhibiting greater sensitivity, underscoring the role of oxygen availability in microbial pollutant tolerance. Taxonomic analysis revealed concentration-dependent shifts, with Actinobacteria and Proteobacteria dominating under aerobic conditions, and Chloroflexi, Bacteroidetes, and Acidobacteria prevailing under anaerobic conditions. The novelty of this study lies in its systematic comparison of microbial community responses and adaptive mechanisms under both aerobic and anaerobic soil conditions, providing critical insights into the differential impacts of PFOA that have previously not been explored.The findings highlight the ecological significance of resilient taxa in sustaining carbon and nutrient turnover in contaminated soils.

## Graphical abstract

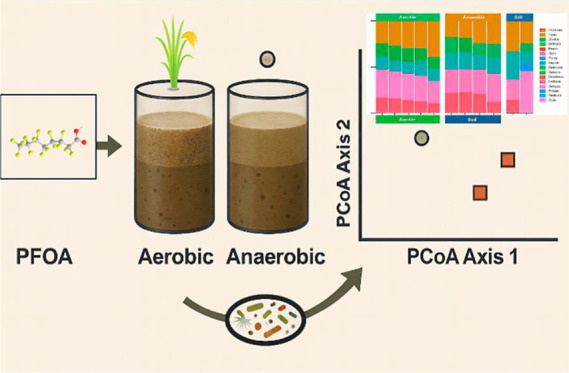

## Introduction

Perfluorooctanoic acid (PFOA) is a synthetic perfluoroalkyl substance widely used in industrial applications due to its exceptional chemical stability and water- and oil–resistant properties.^1,2^ A high level of PFOA has been frequently reported in agricultural soil,^3^ affecting the essential soil microbial community.^4^ The structural diversity among PFAS significantly influences their environmental behavior, persistence, and microbial degradation pathways.^5,6^ For instance, PFOA, with its carboxylic acid functional group (−COOH), exhibits high water solubility,^7,8^ facilitating its transport in aquatic environments. In contrast, PFOS, characterized by a sulfonic acid group (−SO_3_H),^9^ demonstrates greater persistence and bioaccumulation potential due to stronger hydrophobic and lipophobic interactions.^10^ These functional group differences also affect microbial degradation, sulfonic acid groups are generally more recalcitrant to microbial enzymatic processes compared to carboxylic acid groups,^11^ which may undergo partial degradation under specific conditions.

Understanding the interplay between these molecular features and microbial responses is critical for developing effective bioremediation strategies. Microorganisms play a vital role in maintaining soil health by facilitating nutrient cycling, organic matter decomposition, and pollutant breakdown.^12,13^ However, exposure to contaminants such PFOA can inhibit microbial activity, altering soil microbial community composition and function.^14,15^ Previous studies have shown that PFOA can significantly reduce microbial diversity and disrupt metabolic functions, negatively affecting soils under aerobic and anaerobic conditions.^16–18^ Recent field assessments have reported increasing levels of PFOA contamination in agricultural soils, particularly near military sites, wastewater irrigation zones, and biosolid application areas, highlighting the real-world relevance and urgency of understanding its ecological impacts. For instance, studies have shown that elevated concentrations of PFOA can significantly alter microbial diversity and functionality, diminishing populations of beneficial taxa such as Acidobacteria and Proteobacteria that are critical to soil nutrient cycling and organic matter decomposition. Specifically, Huang et al. (2022)^16^ and Xu et al. (2022)^17^ demonstrated that exposure to PFOA under anaerobic conditions severely compromised microbial activity, reducing microbial biomass and shifting community composition toward more pollutant-tolerant but functionally limited groups. Moreover, Chen et al. (2022)^14^ found that exposure to trace amounts of PFOA significantly reduced microbial diversity and disrupted key metabolic pathways in soil microbial communities, highlighting its potency even at low concentrations in altering microbial function essential to ecosystem services. However, the differential responses and adaptive strategies of microbial communities to varying levels of PFOA contamination under both aerobic and anaerobic conditions remain inadequately characterized. Clarifying these microbial responses is essential for predicting soil ecosystem resilience and designing targeted bioremediation approaches. Understanding the specific impacts of PFOA as a function of dose on microbial viability across these conditions is critical for accurately assessing risk and designing effective bioremediation strategies.^19^

This study investigates the effects of PFOA on microbial viability and diversity under irrigated aerobic and flooded anaerobic soils. DNA analysis of the bacterial community in aerobic and anaerobic soils provides insights into the resilience and adaptability of soil bacterial communities under different environmental conditions. This study aims to elucidate the PFOA threshold levels at which microbial activity is compromised, with implications for soil health and PFAS management. The findings will enhance our understanding of microbial tolerance to PFOA and inform future remediation approaches to mitigate PFOA pollution, especially in vulnerable anaerobic soil ecosystems.

## Materials and Methods

### Experimental Design

Soil samples were collected from the top 10 cm of an agricultural field in West Texas. A 10 cm depth was selected because surface soils are the most relevant for direct PFAS exposure from biosolids and irrigation inputs. The soil was classified as a sandy loam based on USDA texture classification, with a maximum water-holding capacity of 28% (w/w). The experimental soil was chosen for its representative physicochemical properties neutral pH, moderate organic matter content, and standard redox potential as previously documented in our earlier report.^20^ Before treatment, soil samples were analyzed for native PFOA contamination, whose levels were 2.26 ng/g, confirming that the collected soil was uncontaminated at baseline. These characteristics make it a suitable model for assessing microbial responses to PFOA under realistic agricultural conditions. . Although this study used a single soil type, the selected soil is characteristic of the region, thereby providing meaningful insights into microbial responses relevant to similar agricultural ecosystems impacted by PFOA contamination. The samples were homogenized and passed through a 2 mm sieve to remove large debris. The sieved soil was preincubated at 25 °C in an incubator for 1 week to stabilize microbial activity.^21^ This preincubation step was performed to ensure consistent microbial conditions in the soil. The physicochemical properties of the collected soils showed oxygen level of 4.8 mg/L, a redox potential (Eh) of +580 mV, and a total organic matter content of 5.2%.

The soil was divided into treatment and control groups. PFOA solutions were prepared to give the final concentrations of 40, 60, 80, and 100 mg/kg, expressed on a dry weight (dw) basis, using deionized water. The concentrations were based on previously reported contamination levels observed near point sources such as industrial discharge sites, landfill leachates, and biosolid-amended soils.^22,23^ Studies have documented that PFOA concentrations in impacted soils can range from tens to hundreds of mg/kg in extreme cases.^24,25^ By using 40–100 mg/kg, we aimed to simulate high-end environmental exposure scenarios to assess microbial sensitivity under stress conditions relevant to contaminated field sites.

### Soil Characterization

The elemental composition and distribution of the soil sample were analyzed using a field emission scanning electron microscope (FE-SEM) equipped with an energy dispersive X-ray spectroscopy (EDS) detector (HITACHI SU3500 under 10^–6^ Torr).^26^ The prepared soil powder was mounted on aluminum stubs using carbon tape and sputter-coated with a thin layer of gold to enhance conductivity. EDS spectra were collected in high vacuum mode at an accelerating voltage of 15 kV. Elemental mapping was conducted on selected regions to visualize the spatial distribution of key elements, including Si, Al, Fe, O, K, Ca, and Mg.

### PFOA Detection in Background Soil

Soil samples were freeze-dried and homogenized to a fine powder using a hand-held grinder. The grinder was rinsed thoroughly with ultrapure water (UPW) and methanol between samples to prevent cross-contamination. Subsamples (0.5 g) were weighed into 50 mL polypropylene (PP) centrifuge tubes. Each sample was extracted with 4 mL of methanol containing 2.0% ammonium hydroxide (HPLC grade). Tubes were vortexed for 2 min, sonicated for 15 min at 30 °C, and shaken for 1 h, followed by centrifugation at 5000 rpm for 30 min. The supernatant was transferred to a clean 15 mL PP tube. This procedure was repeated twice, and the three extracts were combined. The pooled extract was concentrated to approximately 1 mL using a rotary evaporator. Extracts were subjected to cleanup using Supelclean ENVI-Carb cartridges (100 mg, 1 mL capacity; Sigma-Aldrich). Cartridges were preconditioned with 1 mL of methanol followed by 1 mL of UPW. Approximately 1 mL of extract was loaded by gravity flow, and the cartridge was rinsed with 0.5 mL of methanol. The eluate and wash fractions were combined and evaporated under reduced pressure to ∼1 mL. Concentrated extracts were filtered through 0.45 μm syringe filters (glass fiber or PP membrane) into HPLC vials and stored at 4 °C (≤48 h) or −20 °C (long-term) before LC–MS/MS analysis. Shimadzu LC-MS-8050 triple quadrupole liquid chromatography–mass spectrometry was used for the detection of PFOA using EPA Method 1633.

### Soil Sample Preparation

Soil samples (100 g each) were prepared in triplicate for each treatment condition. Two different moisture regimes were employed to simulate aerobic and anaerobic conditions. To create aerobic conditions, 100 g soil was contaminated at each target concentration, and filled with water to approximately 60% of the soil’s field capacity. This volume was chosen to achieve unsaturated conditions, allowing for air exchange within the soil matrix. Samples were then maintained in a loosely capped environment to ensure continuous exposure to atmospheric oxygen. To create anaerobic conditions, each 100 g soil sample at the final PFOA concentration was fully saturated with water to 100% of field capacity. Control samples were prepared without PFOA treatment as baseline references for microbial and DNA analyses. The two moisture conditions were chosen to represent the predominant oxygen availability scenarios encountered in natural and agricultural soils, well-aerated (aerobic) and water-saturated or poorly drained (anaerobic) environments.^27,28^ This distinction is critical as microbial composition and metabolic activity differ significantly between these conditions. While additional moisture gradients could be informative, our focus was on capturing the contrasting microbial responses under the two most ecologically relevant redox conditions to understand how oxygen availability influences PFOA toxicity. All treatments were incubated by shaking at room temperature (∼25 °C) for 7 days. The 7 day experimental period was selected based on prior studies indicating that short-term microbial responses are sensitive and informative indicators of contaminant stress,^27,29^ particularly in the early phase of exposure. Continuous shaking ensured homogeneous exposure of soil particles to PFOA and maintained the defined aerobic or anaerobic conditions by maximizing the interface between soil and solution.

### Microbial Cultivation and Colony-forming Units (CFUs) Counting

After the incubation period, subsamples were collected from each aerobic and anaerobic soil treatment for microbial analysis. No ethical or institutional approval was required for conducting the microbial studies in this research. For aerobic cultivation, 10 μL of each subsample was diluted in sterilized deionized water and spread-plated onto standard nutrient agar, composed of 5.0 g/L peptone, 3.0 g/L beef extract, 15.0 g/L agar, and 1.0 g/L sodium chloride, pH adjusted to 7.0. Plates were incubated aerobically at 28 °C for 48 h in a standard dry incubator (no added CO_2_ or humidity control) and were not sealed (to allow gas exchange). 25 °C represents standard temperature used to promote the recovery and growth rate of mesophilic soil heterotrophs for cfu enumeration. In contrast, bulk soil incubations were conducted at 25 °C to approximate ambient field conditions. The 3 °C difference lies within the mesophilic range and is not expected to shift dominant taxa recovered by culture. Moreover, 16S rRNA community profiling was performed on DNA extracted directly from soils, so plate incubation temperature does not influence the sequencing-based community composition. cfu data are compared only among plates incubated at the same temperature. For anaerobic cultivation, 10 μL of each subsample was similarly diluted in sterilized deionized water and then spread onto reinforced clostridial agar, a medium well-suited for the growth of obligate and facultative anaerobes. RCA consisted of 10.0 g/L peptone, 10.0 g/L beef extract, 3.0 g/L yeast extract, 5.0 g/L glucose, 0.5 g/L l-cysteine hydrochloride (as a reducing agent), and 15.0 g/L agar, with the pH adjusted to 7. Plates were placed in anaerobic jars flushed with nitrogen gas to eliminate oxygen and create a strict anaerobic environment. Incubation occurred at 28 °C for 48 h to facilitate the growth of anaerobic bacteria. Following incubation, bacterial colonies were manually counted using a colony counter, and cfu counts were recorded for each PFOA concentration under aerobic and anaerobic conditions.

### Microbial DNA Extraction and Analysis

DNA was extracted from 0.25 g of soil using the Qiagen DNeasy PowerSoil kit. The V4 region of the 16S rRNA gene was amplified using the universal primer set 515f (5′-GTGCCAGCMGCCGCGGTAA-3′) and 806r (5′-GGACTACHVGGGTWTCTAAT-3′). This region is widely employed for microbial community analysis because it provides high-resolution coverage of bacterial taxa across diverse environments, including soils, while minimizing amplification bias.^30,31^ The DNA concentration was measured using a Qubit Flex Fluorometer (Invitrogen, ThermoFisher Scientific, USA), following Invitrogen’s protocol. Extracted DNA samples were then submitted to the UTEP core facility for high-throughput sequencing on an Illumina MiSeq platform.

### Bioinformatics and Data Analysis

Bioinformatics analysis was conducted using Quantitative Insights into Microbial Ecology (QIIME 2, version 2022.8). Raw sequencing reads were demultiplexed and quality-filtered, followed by denoising and amplicon sequence variant (ASV) generation with DADA2. Taxonomic classification was performed using the SILVA 132 database.

### Statistical Analysis

All experiments were performed in triplicate. Before parametric testing, the normality of residuals was assessed using the Shapiro–Wilk test, and homogeneity of variances was evaluated with Levene’s test; both assumptions were met (*p* > 0.05). Differences between treatments were then analyzed using one-way ANOVA, followed by Tukey’s posthoc test for pairwise comparisons. Correlation analyses were performed using SPSS software (version 19.0, SPSS Inc., USA). Statistical significance was set at a threshold of *P* < 0.05.

## Results and Discussion

### Soil Characterization

Elemental analysis of the soil sample was conducted using Energy Dispersive X-ray Spectroscopy (EDS) to assess its mineralogical composition before experimental design. As shown in Figure 1A, the EDS spectrum revealed the dominant presence of oxygen (O), silicon (Si), and aluminum (Al), indicating a silicate-rich matrix commonly associated with clay minerals such as kaolinite or Illite. Other significant elements include iron (Fe), potassium (K), calcium (Ca), magnesium (Mg), and sodium (Na), which suggest the presence of feldspar and iron oxide minerals. The corresponding elemental mapping in Figure 1. B confirms the spatial distribution of these elements across the soil matrix. Silicon and oxygen are uniformly distributed, supporting the presence of silicate frameworks, while Fe and Al show localized concentrations likely tied to iron oxides and aluminosilicate minerals. The detection of carbon (C) may indicate the presence of organic matter or residual organics from environmental exposure. Overall, the compositional profile reflects a typical mineral soil derived from weathered parent rock material with moderate fertility and buffering capacity.

**Figure 1:**
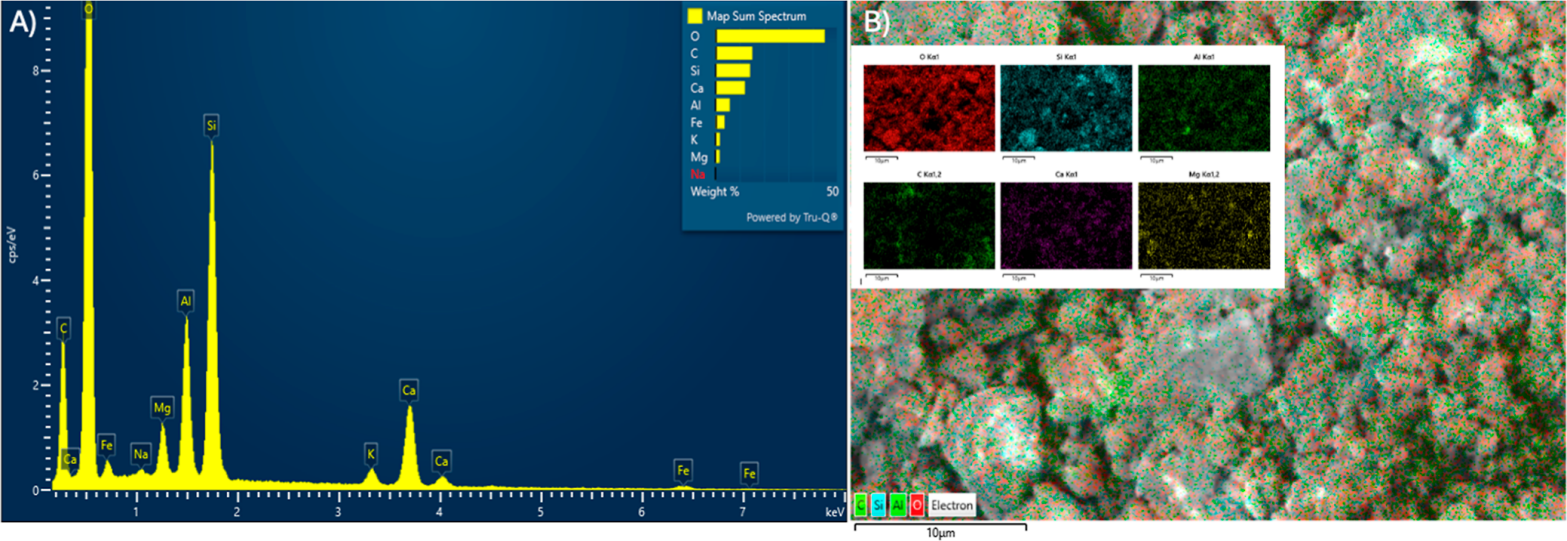
(A) Energy Dispersive X-ray Spectroscopy (EDS) spectrum of the soil sample showing elemental composition, with dominant peaks for O, Si, Al, and Fe. (B) Elemental mapping images displaying the spatial distribution of selected elements (O, Si, Al, C, Ca, Mg, and Fe) across the soil matrix.

### Microbial Viability Analysis in PFOA-Contaminated Aerobic and Anaerobic Soils

As shown in Figure 2A, in aerobic soil conditions, the control group (0 mg/kg PFOA) showed the highest microbial population, achieving a cfu count of approximately 38, as depicted in the figure. As the PFOA concentration in soil increased to 20 mg/kg, there was a statistically significant reduction in cfu, with an observed decrease from 38 colonies per gram of soil in the control group to 32.4 colonies at 20 mg/kg. This trend continued with further reductions at 40 to 100 mg/kg PFOA, with the cfu counts dropping to a minimum of 15.6 in 40 mg/kg. This progressive decline in cfu highlights PFOA’s potent negative impact on microbial viability at concentrations above 20 mg/kg in an aerobic environment.

**Figure 2:**
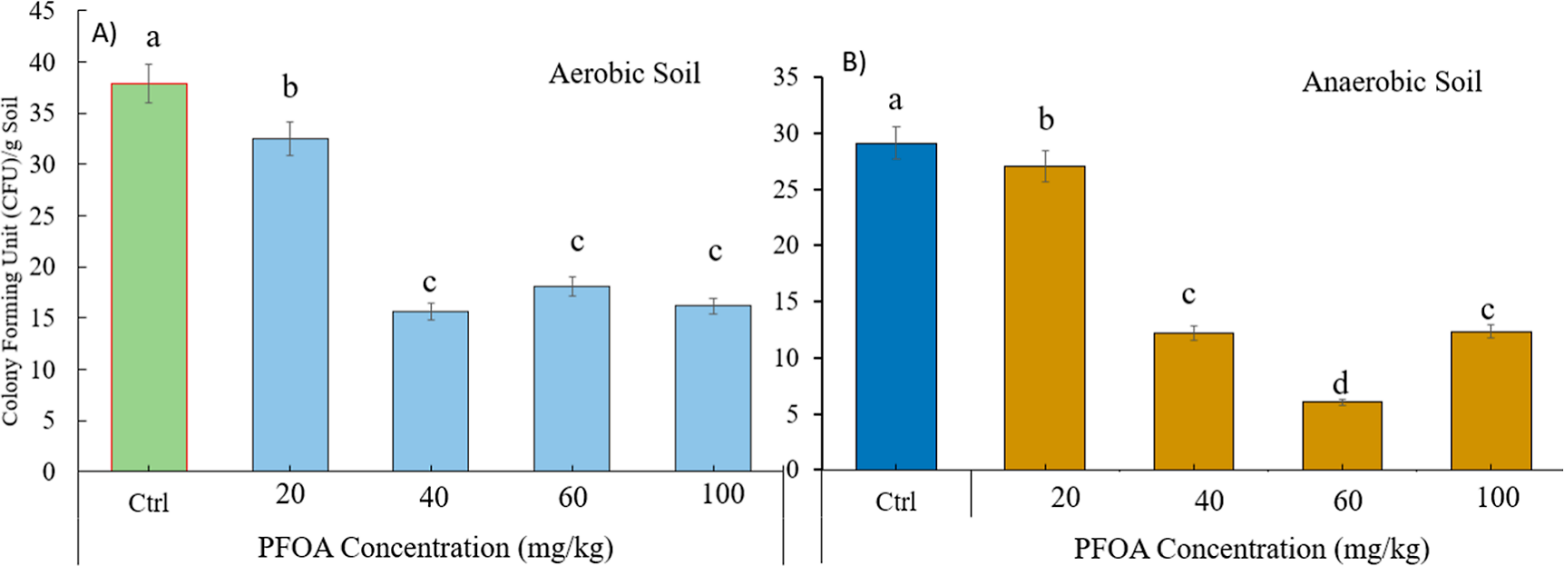
Effect of PFOA concentration on microbial colony-forming units (cfu) in (A) aerobic and (B) anaerobic soil environments. The data points indicate mean cfu counts with standard error bars, and statistical significance is denoted by different letters, analyzed by one-way ANOVA.

As shown in Figure 2B, the cfu pattern in anaerobic soil was similar to aerobic conditions, albeit with a lower starting cfu count in the control group (∼30 cfu). The decrease in cfu was more pronounced at 40 mg/kg, with the most significant reduction observed at 60 mg/kg. At 100 mg/kg, although there was a slight uptick in 12.32 cfu, it remained significantly below the control level, suggesting that PFOA’s toxicity persists even at higher concentrations than 20 mg/kg.

The comparative data between aerobic and anaerobic conditions indicate that microbial populations in anaerobic soil are more sensitive to PFOA exposure. The differential response could be attributed to the inherently greater adaptability of aerobic microbes, which may possess more efficient metabolic or defensive pathways to counteract environmental stressors such as PFOA.^32,33^ In contrast, anaerobic microbial communities may lack these adaptive mechanisms, making them more susceptible to toxic effects.^16,17^

### Microbial Community Structure and Diversity Assessment

The impact of PFOA on soil bacterial communities is illustrated in the heatmap (Figure 3A), which presents the relative abundance of bacterial orders across different PFOA concentrations, treatments, and conditions. Figure 3. B displays the relative abundance of dominant bacterial phyla across the same treatments. As PFOA concentration increased from 20 mg/kg to 60 mg/kg, distinct bacterial orders exhibited variable responses, indicating selective pressures exerted by pollutant levels. Clostridiales and Burkholderiales showed increased abundance at 40 mg/kg, suggesting optimal growth or resilience under moderate pollutant stress.^17,19^ These changes represent observed trends from the sequencing data and were not subjected to formal differential abundance testing. While this suggests potential resilience of these taxa under moderate pollutant stress, confirmation with statistical methods such as ANCOM would be valuable in future analyses. This finding indicates potential adaptive mechanisms, such as pollutant tolerance or metabolic pathways for survival in moderately polluted environments. At 20 mg/kg, Bacillales and Sphingomonadales were prevalent, possibly due to their roles in early stage pollutant degradation or adaptation to low-stress conditions.^34,35^ Bacillales, known for forming spores, may adapt quickly to environmental changes, while Sphingomonadales are associated with organic pollutant degradation, indicating potential activity in breaking down low concentrations of PFOA. Oxygen availability influenced microbial responses to PFOA, as certain bacterial orders adapted to both aerobic and anaerobic conditions. Firmicutes (e.g., Clostridiales) and Gammaproteobacteria (e.g., Burkholderiales) were abundant across both conditions, suggesting versatile metabolic capacities that allow them to withstand redox variation while processing PFOA. This adaptability positions these groups as potential ecological keystones in polluted soils, contributing to community stability in fluctuating oxygen environments. The consistent presence of Firmicutes and Gammaproteobacteria across different treatments highlights their potential ecological roles in maintaining community stability within PFOA-impacted soils. These microbial groups may form mutualistic or commensal relationships within the microbial community, supporting ecosystem resilience against pollutant stress.^36,37^ The observed concentration-dependent shifts in microbial composition indicate that PFOA exerts selective pressures, promoting community adjustments based on microbial resistance and sensitivity. Notably, *Clostridiales* and *Burkholderiales* showed intermediate abundance at 40 mg/kg, suggesting they may possess adaptive mechanisms allowing them to thrive under moderate pollution levels, potentially contributing to bioremediation. In contrast, *Bacillales* and *Sphingomonadales* were prevalent at lower concentrations (20 mg/kg), indicating their role as early responders in pollutant degradation in less contaminated environments. Consistently, *Firmicutes* and *Gammaproteobacteria* were present across both aerobic and anaerobic conditions, highlighting their core role in maintaining community stability^38^ and ecological resilience, likely due to their metabolic flexibility.

**Figure 3:**
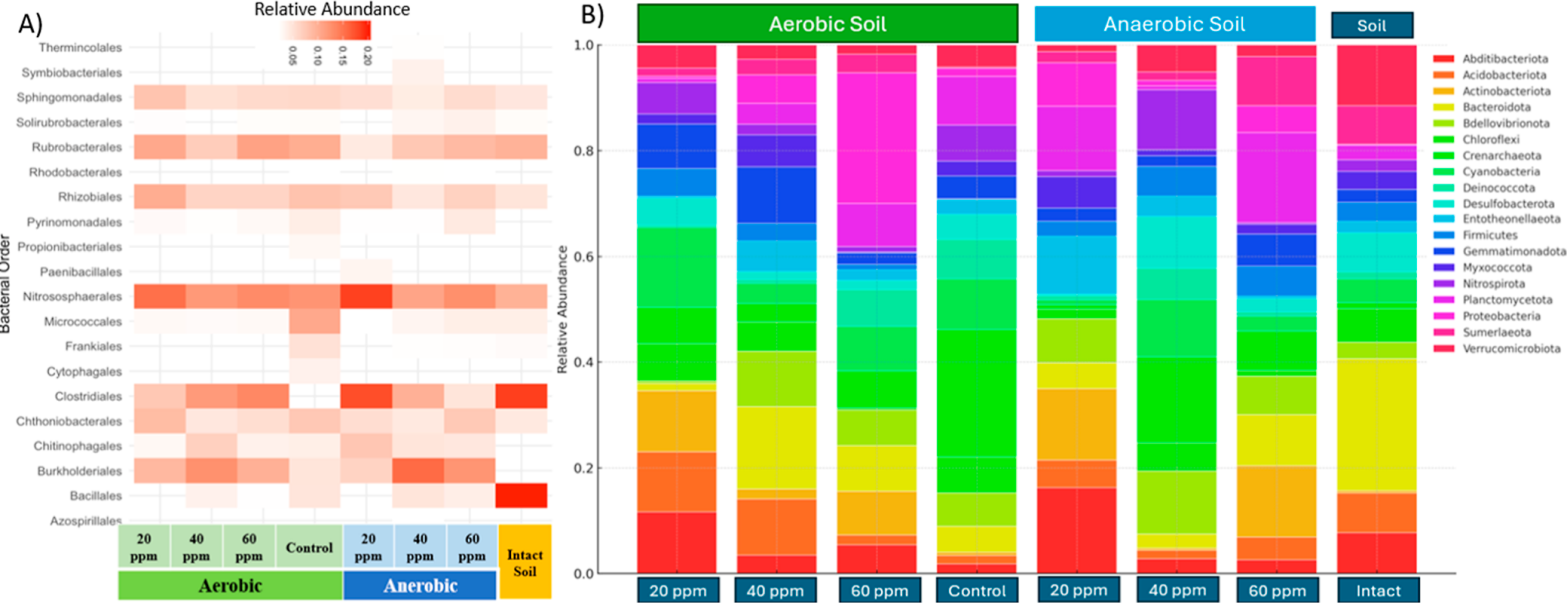
(A) Heatmap showing the relative abundance of bacterial orders across treatments. Increases in certain taxa are based on observed patterns in relative abundance and were not tested for statistical significance. (B) Relative abundance of the dominant bacterial phyla across aerobic, anaerobic, and intact soil treatments at three different PFOA concentrations (20 mg/kg, 40 mg/kg, and 60 mg/kg, including control.

As shown in Figure 3B, in aerobic conditions, Actinobacteria and Proteobacteria emerged as the dominant phyla under aerobic conditions, suggesting a microbial community adapted to oxygen-rich environments. Actinobacteria are known for their role in organic matter decomposition and nutrient cycling,^39^ particularly in soils where they contribute to the breakdown of complex organic compounds such as cellulose and lignin. The prevalence of Proteobacteria, particularly members of the Beta- and Gamma-Proteobacteria classes, aligns with their documented abundance in oxygenated soils and diverse metabolic capabilities.^40^ Proteobacteria are often involved in nitrogen cycling (nitrification and denitrification processes) and play critical roles in biogeochemical cycles,^41^ enhancing soil fertility and structure. This observation corroborates findings from previous studies indicating that Actinobacteria and Proteobacteria thrive in oxygenated conditions,^42,43^ where they facilitate essential ecological processes, including organic matter degradation and nutrient turnover. Under anaerobic conditions, there is an observable shift with Chloroflexi, Bacteroidetes, and Acidobacteria becoming more prominent. This shift reflects an adaptation of these phyla to low-oxygen environments, where they likely contribute to carbon cycling under anaerobic conditions. Chloroflexi are known for their ability to survive in anoxic environments rich in organic carbon.^44^ They play key roles in carbon cycling through processes such as fermentation and anaerobic photosynthesis,^45^ particularly in sediments and subsurface soils. The increased abundance of Chloroflexi in anaerobic conditions is consistent with their known ecological function in low-oxygen, carbon-rich settings. Bacteroidetes also thrive under anaerobic conditions, where they are often involved in the degradation of complex organic compounds and polymers. Their presence suggests an active role in organic matter breakdown and nutrient recycling in oxygen-limited environments, providing energy and nutrients for other microbial populations. Acidobacteria show an affinity for anaerobic conditions as well, which is in line with studies that report their abundance in acidic and nutrient-poor soils. Acidobacteria’s metabolic diversity enables them to survive in low-resource environments,^46^ making them resilient to various stress factors, including reduced oxygen availability.

As shown in Figure 3B, In the control condition (intact soil), *Acidobacteriota* and *Proteobacteria* dominate, reflecting a stable microbial community that is adapted to a variety of environmental conditions. *Acidobacteriota*, which are often associated with nutrient-poor and acidic soils,^47^ suggest the ability to persist in environments with limited resources, contributing to the slow cycling of organic material and carbon sequestration.^47^ *Proteobacteria*, prevalent in the intact soil, underscore their ecological versatility. Their dominance across different treatments, including both PFOA and oxygen variations, highlights their metabolic adaptability and involvement in key biogeochemical cycles such as carbon, nitrogen, and sulfur cycling essential processes for soil health and productivity. Notably, the presence of Beta-, Gamma-, and Delta-Proteobacteria^48^ in both the intact and treated soils indicates their role as ecological generalists. These groups are capable of thriving in both oxygen-rich and oxygen-depleted environments, supporting ecosystem stability and resilience through their metabolic flexibility.

The differential responses to aerobic and anaerobic conditions underscore the influence of oxygen availability on microbial adaptation and PFOA toxicity. The presence of core bacterial groups across oxygen gradients suggests a robust ecological network capable of sustaining community structure despite environmental stressors. These insights could inform targeted bioremediation strategies that leverage the natural resilience of these microbial taxa in managing PFOA contamination.

The relative abundance pertaining to the dynamics of bacterial order is provided in Supporting Information, Figure S1. This data highlights distinct microbial shifts based on environmental oxygen availability and pollutant levels, revealing adaptive responses and ecological roles of key bacterial phyla under different conditions. Along with community shifts, we observed a decrease in unique ASVs (Figure S2), supporting PFOA studies that find decreases in bacterial diversity.^14^

## Ecological Implications and Microbial Functionality

The observed distribution patterns across different conditions and pollutant concentrations provide insights into microbial resilience and functionality in response to environmental stressors. Principal coordinate analysis (PCoA) of Bray–Curtis dissimilarity (Figure 4) illustrates differences between uncontaminated soils and microbial communities affected by PFOA. While the separation among treatments is subtle, the variation in relative abundance at different PFOA concentrations suggests selective pressures that may promote the growth of certain phyla, such as Actinobacteria and Chloroflexi, which are known for their resilience to PFAS in soil.

**Figure 4:**
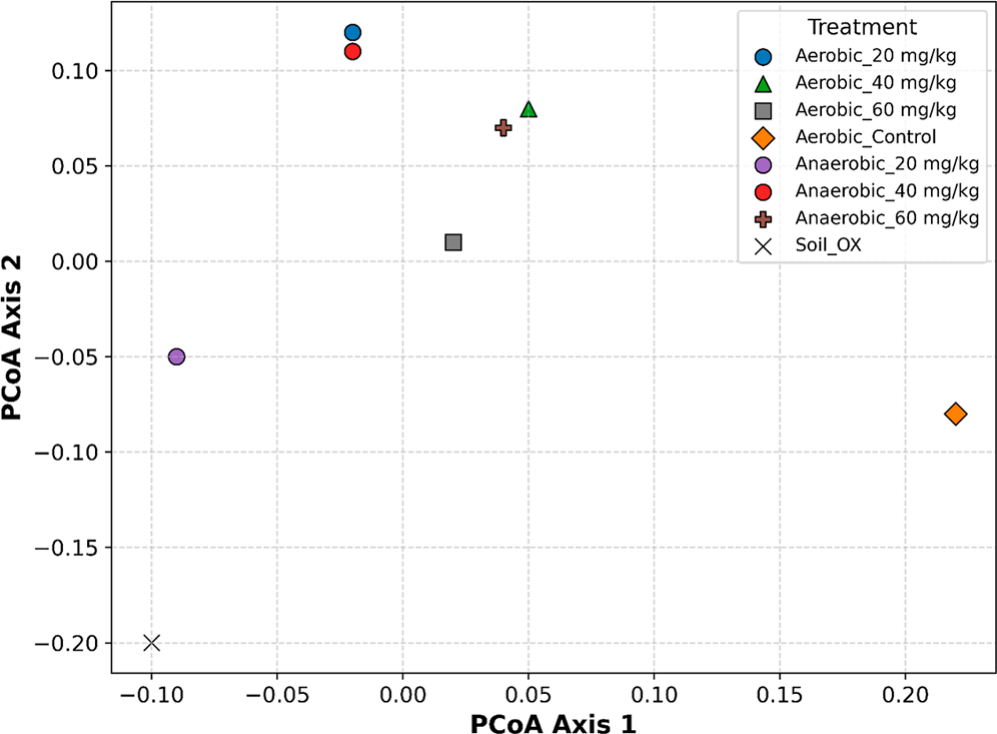
Principal coordinate analysis (PCoA) of Bray–Curtis dissimilarity based on microbial communities across aerobic, anaerobic, and intact soil conditions.

This adaptability highlights the potential role of these microbes in natural bioremediation processes, as they may possess metabolic pathways capable of degrading or tolerating pollutants. The dominance of Proteobacteria and Acidobacteria across treatments underscores their ecological significance in biogeochemical cycles, particularly in carbon and nitrogen processes. The ability of these groups to persist in both aerobic and anaerobic conditions suggests that they are integral to maintaining nutrient availability and soil structure under variable environmental conditions. The diversity metrics are shown in Figure S2, illustrating the impacts of PFOA under different concentrations. Understanding these microbial shifts is crucial for developing strategies for managing contaminated soils. Phyla such as Chloroflexi, Bacteroidetes, and Acidobacteria, which increase in abundance under anaerobic and high-stress conditions, could be targeted in bioremediation efforts to enhance the degradation of persistent pollutants like PFOA.

## Conclusions

This study demonstrates that both PFOA concentration and environmental oxygen availability significantly influence soil microbial community composition and viability. The observed adaptability and resilience of bacterial phyla, such as Actinobacteria, Proteobacteria, and Chloroflexi, underscore their critical roles in maintaining soil health, particularly under conditions impacted by persistent contaminants like PFOA. Actinobacteria and Proteobacteria’s prominence in aerobic conditions aligns with their known capabilities in organic matter decomposition and nutrient cycling, crucial functions that sustain soil productivity. Conversely, the enrichment of *Chloroflexi* under anaerobic conditions highlights their ecological importance in carbon cycling and pollutant resilience in oxygen-limited environments. Members of this phylum are known to survive in anoxic, organic-rich soils and sediments, where they contribute to fermentation and reductive carbon turnover.^45,49^ More recently, metagenomic and enrichment studies have reported that *Chloroflexi* can persist in PFAS-contaminated environments and may harbor reductive pathways relevant to PFAS biotransformation.^32,33^ These findings support our observation of *Chloroflexi* enrichment in PFOA-treated anaerobic soils, underscoring their potential functional role in sustaining microbial activity and mediating pollutant tolerance. Our findings indicate clear concentration-dependent effects of PFOA, with higher concentrations markedly reducing microbial viability and altering community structures. These disruptions pose significant threats to essential soil ecosystem services, including nutrient cycling, organic matter decomposition, and overall soil fertility. From a practical standpoint, these results have critical implications for bioremediation efforts, especially in areas experiencing high levels of contamination, such as military training sites or biosolid-amended agricultural fields. The differential microbial responses observed under aerobic and anaerobic conditions emphasize the need for site-specific remediation strategies tailored to local oxygen dynamics and contamination levels to effectively enhance microbial recovery and facilitate pollutant degradation.

Moreover, the identification of threshold concentrations where microbial activity is severely compromised provides important baseline data for establishing or refining soil quality guidelines and regulatory standards aimed at managing PFOA contamination. Recognizing the functional traits of dominant microbial taxa presents an opportunity to leverage specific microbial groups in bioremediation strategies, potentially optimizing interventions to restore polluted soils.

While this study offers valuable initial insights into microbial community responses to PFOA under controlled laboratory conditions, it also highlights the necessity of broader, long-term investigations, including studies at lower concentration gradients, soil enzyme activities and PFAS residue to bridge laboratory findings with realistic environmental scenarios. Importantly, future in situ experiments conducted in naturally contaminated agricultural soils will be essential for validating our laboratory results, thereby ensuring their broader ecological relevance and applicability to real-world environmental management contexts.

## Supplementary Material



## Notes

Figure S1: Relative abundance of bacterial orders in aerobic, anaerobic, and intact soil treatments at varying pollutant concentrations (20 ppm, 40 ppm, 60 ppm, and control); Figure S2: Diversity Metrics Across Treatments of *Chlorella vulgaris* Exposed to Different Conditions (DOCX)
