# Supplementary material for: Differential Impacts of Perfluorooctanoic Acid (PFOA) on Soil Microbial Communities in Aerobic and Anaerobic Agricultural Soils
Source: ACS Omega. 2025 Sep 19;10(38):44541–8. doi: 10.1021/acsomega.5c06624 (PMC12489684; doi:10.1021/acsomega.5c06624)
Supplement: Supplementary file 1 [file ao5c06624_si_001.docx]

**Differential Impacts of Perfluorooctanoic Acid (PFOA) on Soil Microbial Communities in Aerobic and Anaerobic Agricultural Soils**

Nusrat Easmin^2^, Parikrama Sapkota^3^, Kelly S. Ramirez^3^, Yasaman Mohammadi^1^, Mahesh Narayan^1^, Hamidreza Sharifan*^1,2,4^

*^1^Department of Chemistry and Biochemistry, University of Texas at El Paso, Texas, USA.*

*^2^Environmental Science and Engineering Program, University of Texas at El Paso, Texas, USA.*

*^3^Department of Biological Sciences, University of Texas at El Paso, Texas, USA*

*^4^Sharifarm LLC, Research & Development, El Paso, TX 79911, USA*

[**hsharifan@utep.edu*](mailto:*hsharifan@utep.edu)

**Supplementary Information**

**
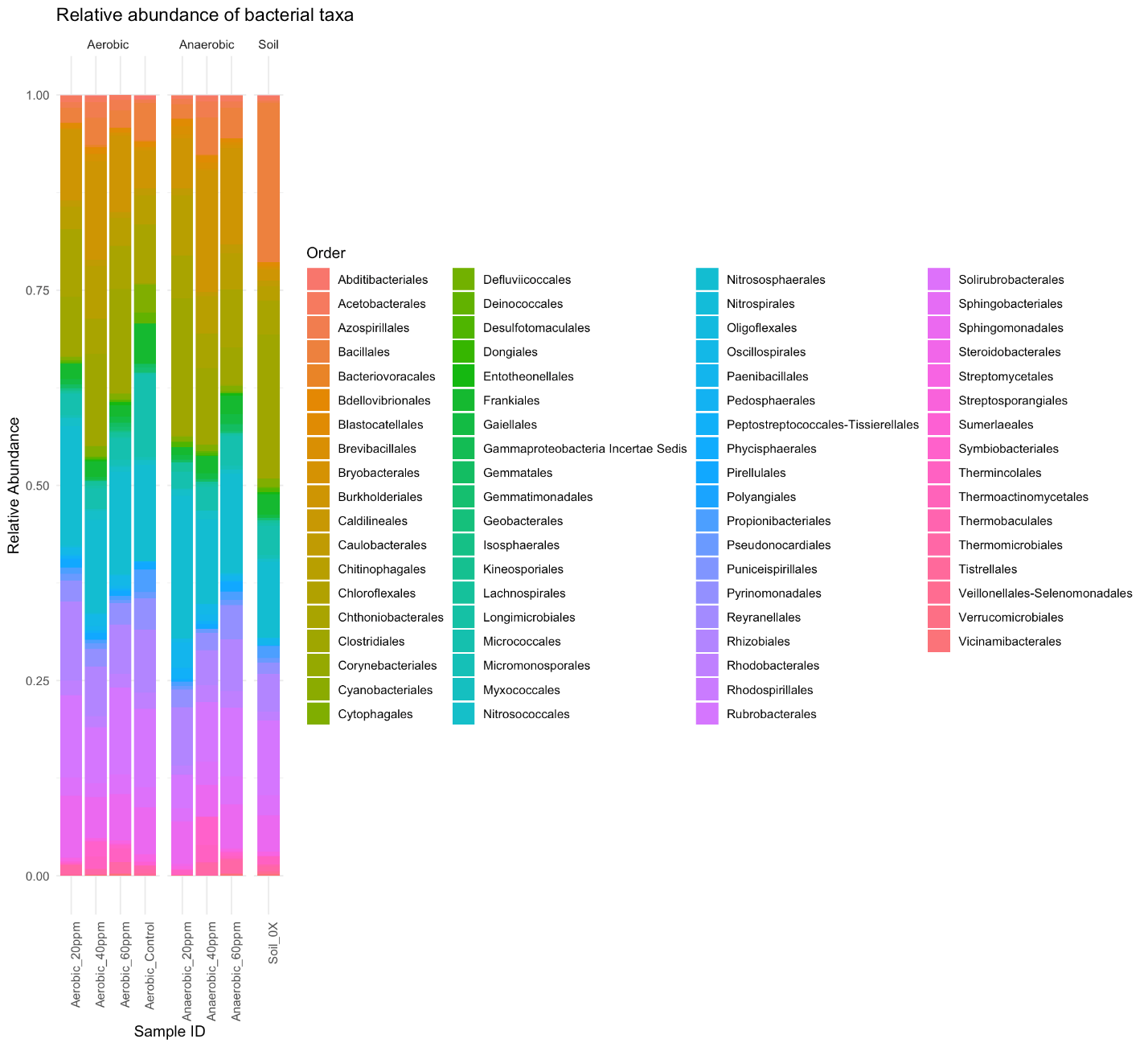
**

Figure S1. Relative abundance of bacterial orders in aerobic, anaerobic, and intact soil treatments at varying pollutant concentrations (20 ppm, 40 ppm, 60 ppm, and control). The stacked bar chart illustrates the diversity of bacterial orders across treatment types and conditions, indicating distinct community compositions for each treatment.


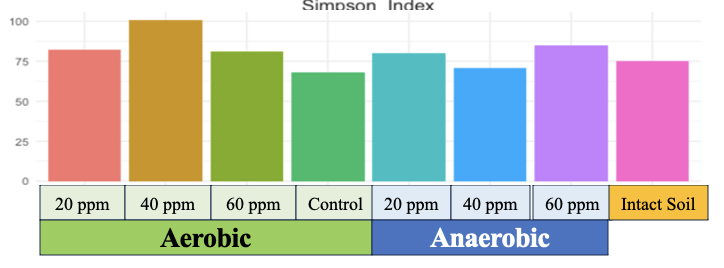


**Figure S3.** Diversity Metrics Across Treatments of *Chlorella vulgaris* Exposed to Different Conditions. The bar plots illustrate Shannon diversity metrics, including unique ASV count (Shannon Index), across various treatments in aerobic, anaerobic, and intact soil environments, with pollutant concentrations at 20 ppm, 40 ppm, 60 ppm, and a control group. Each color represents a specific treatment condition, as indicated in the legend. The Shannon Index reflects species richness and evenness, with the highest diversity observed under aerobic conditions at 40 ppm.
